# Supplementary material for: PAM-Independent Cas12a Detection of Specific LAMP Products by Targeting Amplicon Loops
Source: Int J Mol Sci. 2025 Aug 19;26(16):8014. doi: 10.3390/ijms26168014 (PMC12386220; doi:10.3390/ijms26168014)
Supplement: Supplementary file 1 [file ijms-26-08014-s001.zip › ijms-3794764-supplementary.pdf]

Supplementary Materials

for

PAM-independent Cas12a detection of specific LAMP products by targeting amplicon loops

Konstantin G. Ptitsyn [a], Leonid K. Kurbatov [a], Svetlana A. Khmeleva [a], Daria D. Morozova [a], Elena V. Suprun [b], Sergey P. Radko [a], Andrey V. Lisitsa[1]

[a] Institute of Biomedical Chemistry, Pogodinskaya Street, 10/8, Moscow, 119121 Russia

[b] Chemistry Faculty of M.V. Lomonosov Moscow State University, Lenin Hills, 1/3, Moscow, 119991 Russia

Corresponding author: E-mail: radkos@yandex.ru (Sergey P. Radko)

Table S1. The list of strains used in the study. Strains are from the All-Russian Collection of Microorganisms (VKM), the German Collection of Microorganisms and Cell Cultures (DSMZ) and the Collection of the All-Russian Scientific Research Institute of Phytopathology (VNIIF).

**Species**	**Host**	**Origin**	**Collection**	**Strain Number**
Clavibacter sepedonicus	potato	USA	VKM	Ac-2753
Clavibacter sepedonicus	potato	USA	VKM	Ac-1405
Clavibacter michiganensis	tomato	Zambia	VKM	Ac-1144
Clavibacter michiganensis	tomato	USA	VKM	Ac-1403
Clavibacter phaseoli	common beans	Spain	VKM	Ac-2641
Clavibacter insidiosus	alfalfa	USA	VKM	Ac-1402T
Clavibacter nebraskensis	maize	USA	VKM	Ac-1404T
Clavibacter tesselarius	wheat	USA	VKM	Ac-1406T
Dickeya chrysonthemi	Chrysanthemum morifolium	USA	DSMZ	DSM 4610
Dickeya solani	potato	Russia	VNIIF	1C3
Pectobacterium versatile	potato	Russia	VKM	B-3416
Pectobacterium aquaticum	potato	Russia	VKM	B-3417
Pectobacterium polaris	potato	Russia	VKM	B-3420
Pectobacterium parmentieri	potato	Russia	VKM	B-3423
Pectobacterium carotovorum	potato	Denmark	VKM	B-1247
Pectobacterium brasiliensis	potato	Russia	VKM	B-3424
Pectobacterium brasiliensis	potato	Russia	VKM	B-3425
Pectobacterium odoriferum	potato	Russia	VNIIF	1557
Escherichia coli	clinical isolate	USA	VKM	B-3034

Table S2. Sequences of DNA oligonucleotides used in the study. t_gRNA-B, t_gRNA-F-14, t_gRNA-F-16, t_gRNA-F-18, t_gRNA-F-20, and t_gRNA-F-22 – DNA templates for enzymatic synthesis of gRNAs. FAM – 6-carboxyfluorescein, ROX - carboxy-X-rhodamine, BHQ1 – Black Hole Quencher 1, BHQ2 – Black Hole Quencher 2.

Name	Sequence (5′→ 3′)
FIP*	TCTGAGTCGGACGCGCTCCGTGTGGCGGAGGAGGAA
BIP*	CAAAGCGCCCCTCCAGCTTCTACGGGTTCATCGCCCTC
F3*	ACCGTCTCCTTGATGGAGTG
B3*	GCCGAACCTCTGGGTGT
LF*	CGCATCATCGTCGAGAACGT
LB*	CAGGAGGCTCAGGAGCGAGA
t_gRNA-B	TCTCGCTCCTGAGCCTCCTGATCTACAACAGTAGAAATTCCCCTAT AGTGAGTCGTATTA
t_gRNA-F-14	TGCGCACGTTCTCCATCTACAACAGTAGAAATTCCCCTATAGTGA GTCGTATTA
t_gRNA-F-16	TGCGCACGTTCTCCACATCTACAACAGTAGAAATTCCCCTATAGT GAGTCGTATTA
t_gRNA-F-18	TGCGCACGTTCTCCACGAATCTACAACAGTAGAAATTCCCCTATA GTGAGTCGTATTA
t_gRNA-F-20	TGCGCACGTTCTCCACGATGATCTACAACAGTAGAAATTCCCCTA TAGTGAGTCGTATTA
t_gRNA-F-22	TGCGCACGTTCTCCACGATGATATCTACAACAGTAGAAATTCCCC TATAGTGAGTCGTATTA
T7P	TAATACGACTCACTATAGGG
FAM-MR	FAM-TTATT-BHQ1
ROX-MR-5	ROX-TTATT-BHQ2
ROX-MR-8	ROX-TTATTATT-BHQ2

* LAMP primers – inner primers FIP and BIP, outer primers F3 and B3, and loop primers LF and LB

Table S3. The arithmetic means of characteristic amplification time (t_c), the corresponding standard deviations, and the reciprocal of t_c ($1/t_c$) for LAMP of *C. sepedonicus* genomic DNA both in absence and presence of potato DNA, based on results of 3 independent experiments. *p*-Values are provided for pairwise comparisons of t_c values in absence and presence of potato DNA. The *p*-values were calculated by the two-tailed Student's test (*t*-test), using the Microsoft Excel program package. The reciprocals of mean t_c value ($1/t_c$) are provided for convenience of discussion.

DNA load (genome copies per reaction)	no potato DNA		potato DNA (200 ng per LAMP reaction)		*p*-value
	t_c (min)	$1 / t_c$ (min^{-1})	mean t_c	$1 / t_c$ (min^{-1})	
10	17.1 ± 2.4	0.058 ± 0.007	20.6 ± 2.5	0.049 ± 0.006	0.220
100	14.9 ± 1.1	0.067 ± 0.005	17.4 ± 1.2	0.057 ± 0.005	0.102
1000	14.1 ± 0.9	0.071 ± 0.004	14.3 ± 1.0	0.070 ± 0.005	0.838
10000	12.3 ± 0.3	0.081 ± 0.002	12.9 ± 0.3	0.078 ± 0.002	0.103

Table S4. The results of examining DNA preparations extracted from potato tuber samples contaminated and not contaminated with *C. sepedonicus* (strain Ac-1405) by real-time PCR and the LAMP/Cas12a detection system. The result of real-time PCR is presented as an average value of the cycle threshold (C_t) for duplicate measurements; the result of the LAMP/Cas12a detection is shown as positive (+) or negative (–), based on kinetics of FAM-MR-5 cleavage as in Fig. 2A. LAMP is conducted with a load of 200 ng of extracted DNA (1 µL of DNA sample) for 30 min. Real-time PCR is carried out for 50 cycles, using the commercial kit for *C. sepedonicus* detection. 5 µL of DNA sample were loaded per PCR reaction as recommended by the kit manufacturer. The number of *C. sepedonicus* genome copies per µL of sample was determined with the standard curve provided by the manufacturer of the kit. ND – not defined.

**Potato sample type**	C_t	**genome copies per µL of DNA sample**	**LAMP/Cas12a detection**
contaminated with *C. sepedonicus*	26.6	25100	+
	30.0	2510	+
	33.6	251	+
	36.7	25	+
	40.2	2.5	–
not contaminated with *C. sepedonicus*	ND	0	–
	ND	0	–
	ND	0	–

Table S5. The p-values of pairwise comparisons of V_0 values for *C. sepedonicus* strains Ac-1405 and Ac-2753 with those for other tested strains of *Clavibacter* species. The p-values were calculated by the two-tailed Student's test (t-test), using the Microsoft Excel program package. The p-value above 0.05 is highlighted by red fonts.

C. sepedonicus	**gRNA**	***C. nebraskensis***	***C. michiganensis***		***C. insidiosus***	***C. phaseoli***	***C. tesselarius***
			**Ac-1144**	**Ac-1403**			
**Ac-1405**	**gRNA-F-16**	0.00094	0.00157	0.00087	0.00179	0.00233	0.00142
	**gRNA-F-18**	0.00006	0.00011	0.00003	0.00003	0.00004	0.00003
	**gRNA-F-20**	0.00003	0.00008	0.00009	0.00006	0.00009	0.00042
	**gRNA-F-22**	0.01923	0.00019	0.00074	0.00044	0.00031	0.00025
	**gRNA-F-24**	0.00842	0.00211	0.01437	0.01073	0.00899	0.01495
**Ac-2753**	**gRNA-F-16**	0.00184	0.00267	0.00167	0.00314	0.00375	0.00252
	**gRNA-F-18**	0.00073	0.00998	0.00039	0.00041	0.00034	0.00034
	**gRNA-F-20**	0.00003	0.00013	0.00015	0.00009	0.00015	0.00051
	**gRNA-F-22**	0.16421	0.00015	0.00044	0.00026	0.00017	0.00008
	**gRNA-F-24**	0.00241	0.00234	0.00616	0.00444	0.00287	0.00678

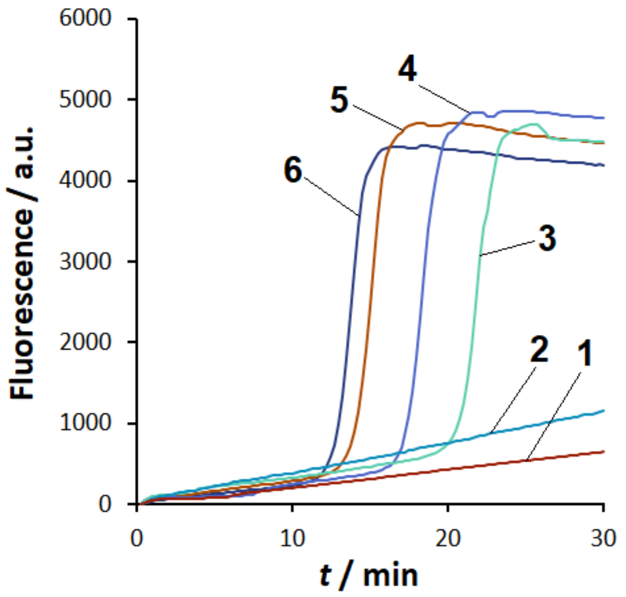

Figure S1. Representative amplification curves. LAMP is conducted with *C. sepedonicus* genomic DNA (stain Ac-1405, Table S2) in the presence of 200 ng of potato DNA. Curve 1 – NTC (no template control), curves 2, 3, 4, 5, and 6 – *C. sepedonicus* DNA loads of 3.7 fg, 37 fg, 370 fg, 3.7 pg, and 37 pg per reaction, respectively (corresponds to 1, 10, 100, 1000, and 10000 copies of genome per reaction).

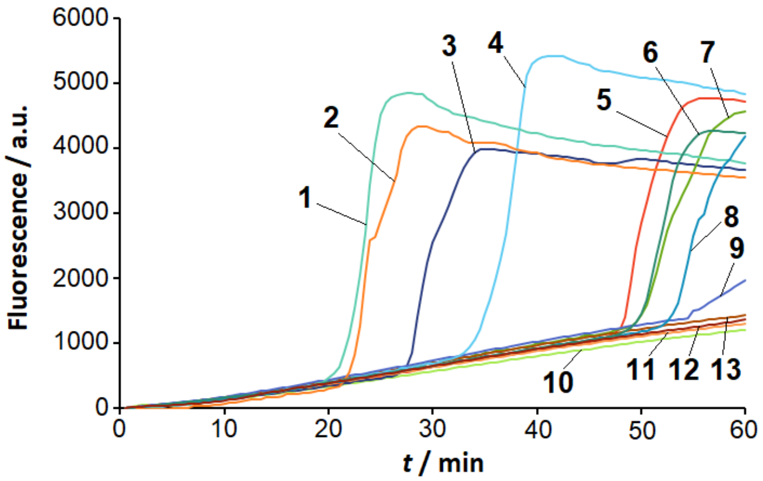

Figure S2. Representative amplification curves. LAMP is conducted with genomic DNA of *Clavibacter*, *Pectobacterium*, *Dickeya*, and *Escherichia* species (Table S2). Curves 1 and 2 – strains Ac-2753 and Ac-1405 of *C. sepedonicus*, respectively, with DNA loads of 37 fg (10 copies of genome) per reaction; the curves are presented for reference purposes. Curves 3 to 13 – strains 1557, B-1247, B-3424, 1C3, B-3416, DSM 4610, B-3423, B-3034, B-3425, B-3420, and B-3417, respectively (Table S1); DNA loads of 37 pg per reaction.

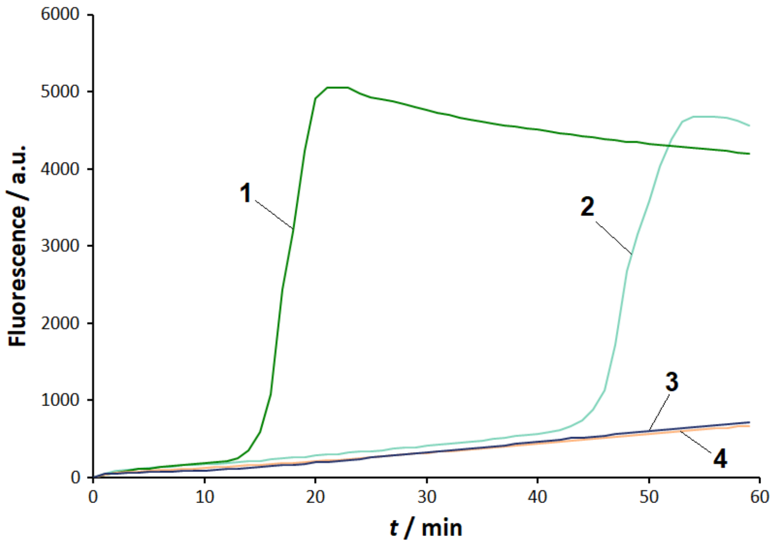

Figure S3. Representative amplification curves. LAMP is conducted with *C. sepedonicus* genomic DNA (stain 2753, Table S2) with DNA load of 370 fg (100 copies of genome) per reaction. Curves 1 and 2 – LAMP in the presence and absence of loop primers. respectively; curves 3 and 4 – the corresponding NTCs (no template controls).

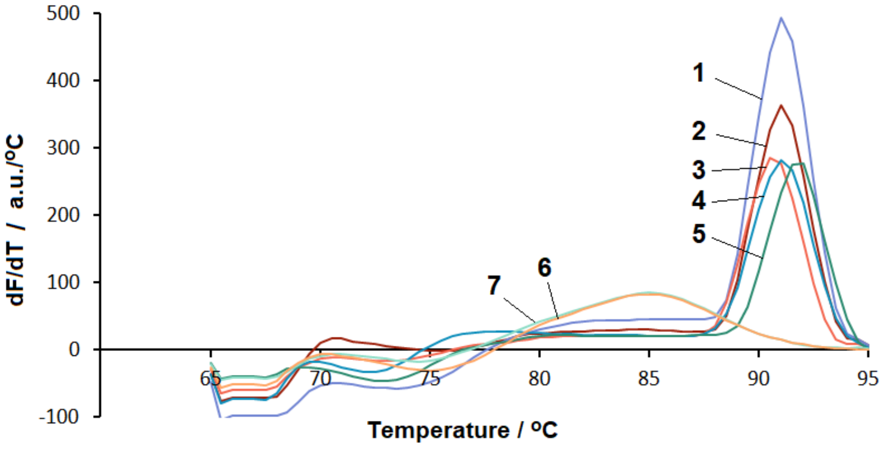

Figure S4. Melting curve analysis of LAMP products. Curves 1, 2, 3, 4, and 5 – LAMP products obtained with genomic DNA of *C. sepedonicus* (strains Ac-2753 and Ac-1405), *D. solani* (strain 1C3), *P. carotovorum* (strain B-1247), and *P. brasiliensis* (strain B-3424), respectively. Curves 6 and 7 – NTC LAMP. LAMP was conducted as in Figure S2. Melting was carried out on a CFX-96 thermal cycler (Bio-Rad Laboratories, Hercules, CA, USA), using EvaGreen as a fluorescent dye.

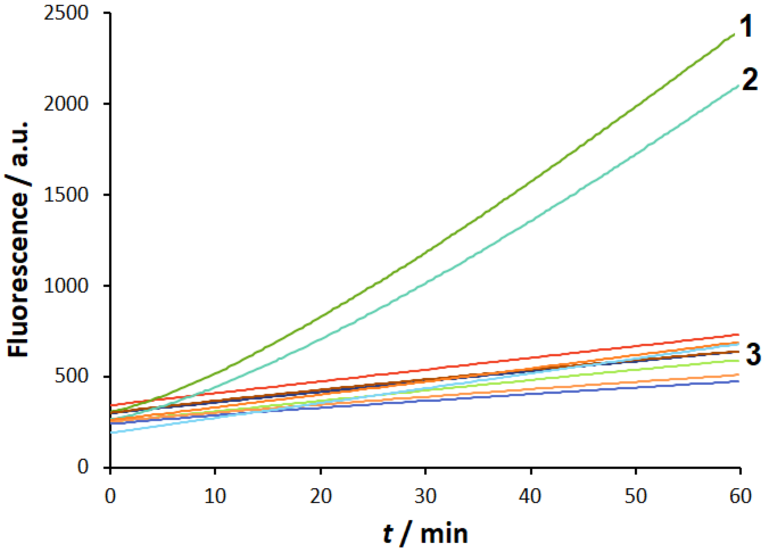

Figure S5. Representative kinetic curves of MR cleavage by the activated Cas12a nuclease. 50 µL of Cas12a reaction mixture containing 60 nM of Cas12a/gRNA-F-16 complex, 1 µM of FAM-MR, and 6 mM Mg^{2+} ions. Curves 1 and 2 – 1 µL of the completed LAMP reaction with 37 fg of *C. sepedonicus* genomic DNA per reaction (stains Ac-1405 and Ac-2753, Table S2). respectively. Curves marked by 3 represent experiments conducted with 1 µL of the completed LAMP reaction with 37 fg of genomic DNA per reaction for other *Clavibacter* species tested (Table S2), as well for NTC LAMP and the control (LAMP buffer instead of completed LAMP reaction).

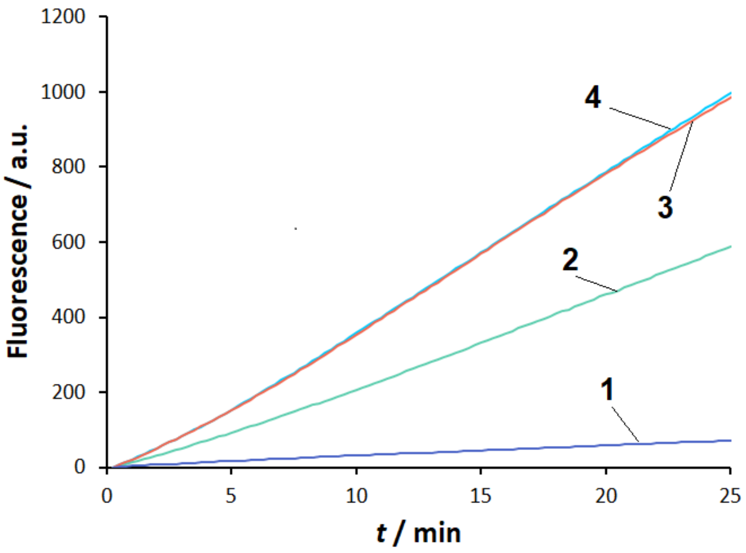

Figure S6. Representative kinetic curves of ROX-MR cleavage by the activated Cas12a nuclease. 10 µL of the completed LAMP reaction with 37 fg of *C. sepedonicus* genomic DNA (stain Ac-2753. Table S2) per reaction. 50 µL of Cas12a reaction mixture containing 60 nM of Cas12a/gRNA-B complex, 1 µM of ROX-MR-5 and various concentrations of Mg^{2+} ions. Curves 1, 2, 3, and 4 correspond to 6, 9, 12, and 18 mM of Mg^{2+} ions, respectively.

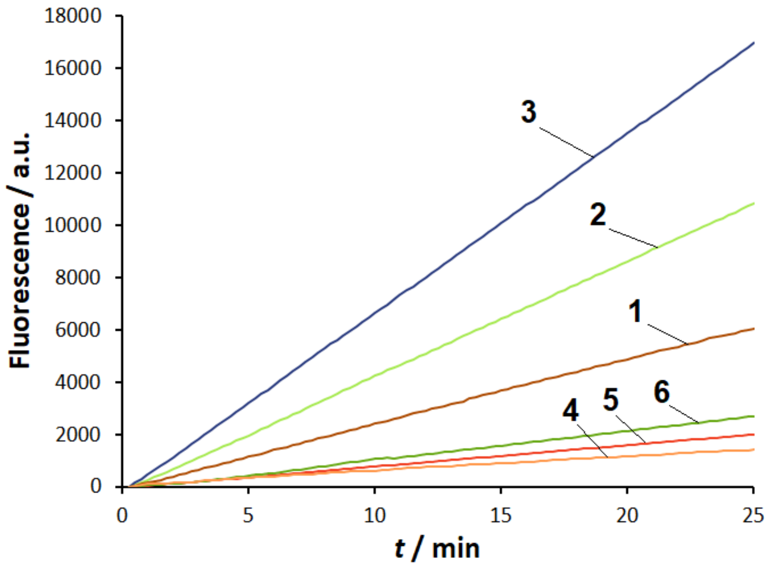

Figure S7. Representative kinetic curves of ROX-MR cleavage by the activated Cas12a nuclease. The conditions as in Figure S5, except for that the concentration of Cas12a/gRNA-B complex varied, the concentration of Mg^{2+} ions was 18 mM, and the concentration of ROX-MR-5 was 18 μM. Curves 1, 2, and 3 – 60, 120, and 180 nM of Cas12a/gRNA-B complex, respectively; curves 4, 5, and 6 – the corresponding controls.

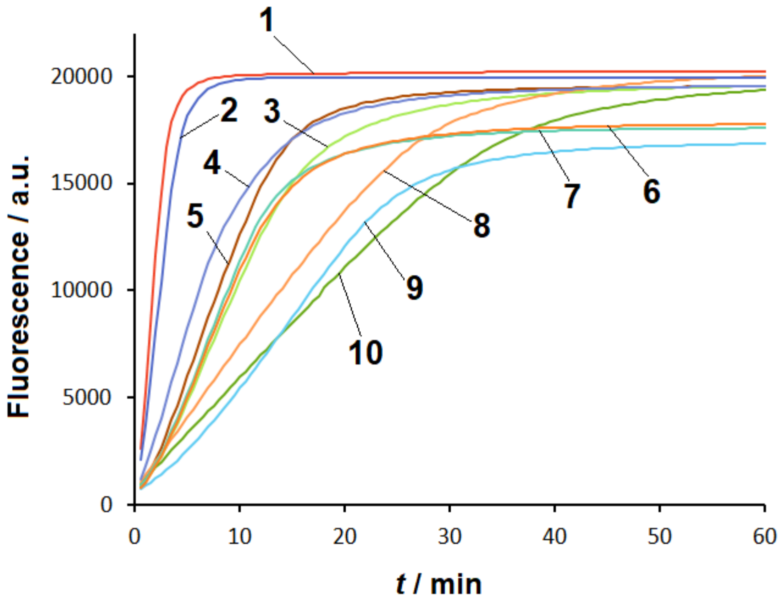

Figure S8. Representative kinetic curves of ROX-MR-8 cleavage by the activated Cas12a nuclease. Concentrations of Cas12a/gRNA-F-20 complex, Mg^{2+} ions, and ROX-MR-8 were 180 nM, 18 mM, and 18 μM, respectively. Other conditions as in Figure S5. Curves 1 to 10 – *C. sepedonicus* (strains Ac-2753 and Ac-1405), *C. insidiosus*, *C. nebraskensis, C. michiganensis* (strain Ac-1403), *C. phaseoli*, *C. michiganensis* (strain Ac-1144), *C. tessellarius*, NTC LAMP, and control, respectively.
